# A Case of Skin Disease—Pediculus Corposis

**Published:** 1891-10

**Authors:** John Hall

**Affiliations:** Victoria, B. C.; For the Canadian Institute of Homœopathy


### A CASE OF SKIN DISEASE—PEDICULUS CORPOSIS.

JOHN HALL, M. D., VICTORIA, B. C.

(For the Canadian Institute of Homœopathy.)

The following case occurred in the year 18— on a lady in the upper walks of life having dark hair, a full habit, aged about fifty, of most cleanly habits, washing repeatedly, and mother of five children.

The allopathic diagnosis of the disease is given because of its *apparent* character, not that I agree with them in their pathological name. And it having existed a long time was subjected to the treatment which that school so heavily resort to in such cases; fortunately for the patient only partially successful; the so-called pediculé returning again and again after temporary death or destruction after each treatment.

The case is, however, given only from memory, the records of it with all others remaining in Toronto. Consequently the diagnosis of the remedy cannot be fully recalled, but the malady having a direct bearing against the teachings of the old school, who insist that such ailments are always the *cause* of disease existing only in the habits of the patients, is taken from among similar ones, as illustrating how all *true* homœopaths recognize and treat them. The writer living among those who with the late Dr. Guernsey and others think less and less of the diagnosis, while in harmony with that equally eminent physician, the late Dr. Lippe, in bestowing intense care in the study and diagnosis of the remedy, a study which often requires an extraordinarily painstaking procedure.

In the malady given the patient was, as before said, in the upper walks of life, and of most cleanly habits, having resorted to those means which with others are unfortunately only too often successful in *suppressing* such outward manifestations. But taking all into consideration, all her antecedents and present condition, the conclusion was arrived at that she was suffering from a deeply seated dyscrasia of which the creeping things on the flesh were merely an outcome, and so treating her accordingly—not giving all her symptoms, for the reasons already assigned, recalling only the remedy *Lycopodium*, which has given help, and in *very rare doses* effectually curing the disease in a few months, and, like all true cures, endowing her with perfect health as well.

Here let me remark that so long as our school is governed exclusively in its diagnosis by that of the dominant one we shall often flounder in darkness, not finding the truth, though it may be necessary at times that some diseases be known or recognized by their pathological names certainly as seldom guiding us in their treatment. Indeed we may truly though painfully add that these things are frequently hidden from the wise and prudent, but revealed unto lambs: those having the childlike and teachable spirit.

